# Protein encapsulation: a new approach for improving the capability of small-molecule fluorogenic probes†

**DOI:** 10.1039/c9sc03961a

**Published:** 2019-11-27

**Authors:** Hai-Hao Han, Adam C. Sedgwick, Ying Shang, Na Li, Tingting Liu, Bo-Han Li, Kunqian Yu, Yi Zang, James T. Brewster, Maria L. Odyniec, Maria Weber, Steven D. Bull, Jia Li, Jonathan L. Sessler, Tony D. James, Xiao-Peng He, He Tian

**Affiliations:** Key Laboratory for Advanced Materials, Joint International Research Laboratory of Precision Chemistry and Molecular Engineering, Feringa Nobel Prize Scientist Joint Research Center, School of Chemistry and Molecular Engineering, East China University of Science and Technology 130 Meilong Road Shanghai 200237 P. R. China xphe@ecust.edu.cn; Department of Chemistry, University of Bath Bath BA2 7AY UK chstdj@bath.ac.uk; National Center for Drug Screening, State Key Laboratory of Drug Research, Shanghai Institute of Materia Medica, Chinese Academy of Sciences 189 Guo Shoujing Rd. Shanghai 201203 P. R. China jli@ecust.edu.cn; Department of Chemistry, University of Texas at Austin 105 E 24th Street A5300 Austin TX 78712-1224 USA sessler@cm.utexas.edu; National Facility for Protein Science in Shanghai, Zhangjiang Laboratory Shanghai 201210 P. R. China; Center for Supramolecular Chemistry and Catalysis, Department of Chemistry, Shanghai University 99 Shang-Da Road Shanghai 200444 P. R. China

## Abstract

Herein, we report a protein-based hybridization strategy that exploits the host-guest chemistry of HSA (human serum albumin) to solubilize the otherwise cell impermeable ONOO^−^ fluorescent probe **Pinkment-OAc**. Formation of a **HSA**/**Pinkment-OAc** supramolecular hybrid was confirmed by SAXS and solution-state analyses. This **HSA**/**Pinkment-OAc** hybrid provided an enhanced fluorescence response towards ONOO^−^*versus***Pinkment-OAc** alone, as determined by *in vitro* experiments. The **HSA**/**Pinkment-OAc** hybrid was also evaluated in RAW 264.7 macrophages and HeLa cancer cell lines, which displayed an enhanced cell permeability enabling the detection of SIN-1 and LPS generated ONOO^−^ and the *in vivo* imaging of acute inflammation in LPS-treated mice. A remarkable 5.6 fold (RAW 264.7), 8.7-fold (HeLa) and 2.7-fold increased response was seen relative to **Pinkment-OAc** alone at the cellular level and *in vivo*, respectively. We anticipate that HSA/fluorescent probe hybrids will soon become ubiquitous and routinely applied to overcome solubility issues associated with hydrophobic fluorescent imaging agents designed to detect disease related biomarkers.

## Abstract 2

Herein, we report a protein-based hybridization strategy that exploits the host–guest chemistry of HSA (human serum albumin) to solubilize the otherwise cell impermeable ONOO^−^ fluorescent probe **Pinkment-OAc**.

## Graphical abstract

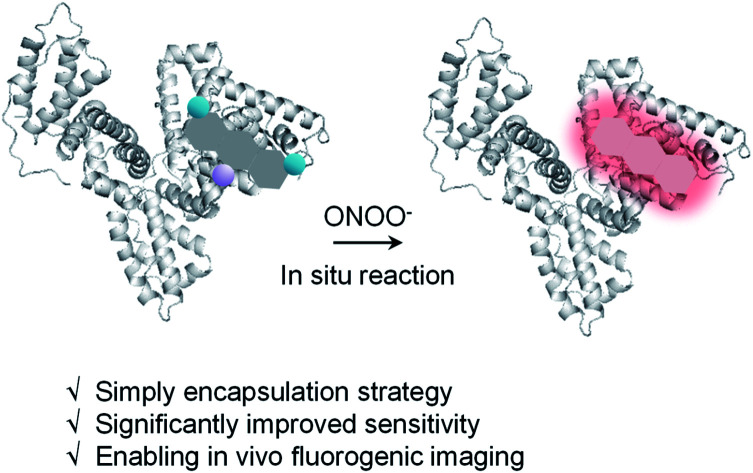

## Introduction

**Scheme 1:**
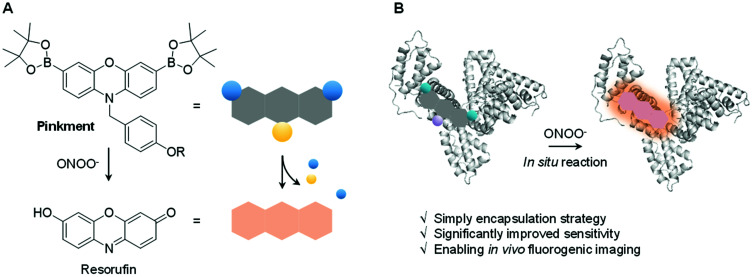
(A) Reaction mechanism of **Pinkment-OAc** (R = Ac) and **Pinkment-OH** (R = H) in the presence of ONOO^−^. (B) Human serum albumin (HSA)-encapsulation of **Pinkment** enabling the detection of ONOO^−^*in vitro* and *in vivo*.

Biochemical processes involve complex interactions between cellular components. Signalling molecules such as reactive oxygen species and reactive nitrogen species (ROS/RNS) play a crucial role in mediating these interactions and are required to maintain proper cell function. ROS/RNS consist of oxidative oxygen and nitrogen-based ions or free radicals.^1,2^ The overexpression of ROS/RNS has been implicated in an array of diseases, including cancer, inflammatory diseases, and CNS neurodegeneration.^3,4^ The widespread interest in biochemical processes where ROS/RNS play key roles, as well as deviations from homeostasis associated with various disease states, has provided an inspiration to develop probes that allow for the facile detection of these species. Fluorescence-based probes have received considerable attention in recent years in the context of ROS/RNS sensing. Ground-up synthetic methods have, for instance, allowed for the incorporation of reactive functionalities within the fluorophore scaffolds used to create such probes.^5–9^ These modifications have conferred utility for the detection and visualization of aberrant ROS/RNS levels considered causal in certain diseases.^6–8^ Despite significant progress, a number of issues remain to be addressed in the area of ROS/RNS probe design. In particular, standard fluorescent probes are often hydrophobic leading to poor cell permeability and aggregation induced quenching (ACQ).^10^ To overcome these limitations, a number of supramolecular-based strategies have been developed.^11–17^ Complementary to these efforts, researchers have utilized protein-based hybridization strategies (host–guest assembly) as a means to overcome the poor solubility and enhance the imaging capability of a contrast agent used in bioimaging applications.^18,19,39^ We thus envisaged that judicious design of an appropriate fluorescent probe–protein complex might permit enhanced sensitivity towards the detection of a disease-specific biomarker. Herein, we present the first demonstration of a fluorescent probe–protein complex strategy that results in the enhanced *in vitro* and *in vivo* detection of a key ROS/RNS signaling agent, in this case peroxynitrite (ONOO^−^) – Scheme 1.

ONOO^−^ is a RNS that is formed *via* the diffusion-controlled reaction between superoxide (O_2_˙^−^) and nitric oxide (NO˙). ONOO^−^ is characterized by its deleterious properties, causing irreversible damage to a range of biological targets.^40^ Recent efforts have focused on the development of fluorescent probes for the detection of ONOO^−^.^20–24^ Towards this end, boronates have proven useful, allowing for a “turn-on” mechanism when incorporated within the sensing motif. The inherent reactivity of these substituents confers selectivity towards ONOO^−^ over other ROS signaling molecules, such as hypochlorous acid (HOCl/ClO^−^) and hydrogen peroxide (H_2_O_2_).^25^ Recently, we developed a series ‘**Pinkment**’ fluorescent probes based on resorufin.^26^ We designed those ‘**Pinkment**’ probes to display ‘AND’-logic in the presence of two different biologically important species.^26^ In particular the **Pinkment-OAc** probe was designed to be activated by an esterase ‘AND’ H_2_O_2_ but the probe exhibited poor solubility and low cell permeability limiting its biological applications. Our aim with this research was to develop a general strategy to improve the sensitivity, solubility and uptake capabilities of a poorly performing fluorescent probe without changing its structure. Since, **Pinkment-OAc** could be selectively activated by ONOO^−^ (*via* oxidation of the boronate and cleavage of the acetate ester – Scheme S1†), it represented a good example of an underperforming probe, suitable for development as an ONOO^−^ selective fluorescent probe–protein hybrid with enhanced biological applicability.

Therefore, **Pinkment-OAc** was synthesized using the previously reported procedure.^26^ Incorporation of **Pinkment-OAc** within HSA to give **HSA**/**Pinkment-OAc** was then achieved *via* sonication of a simple mixture of the probe and HSA in water. In addition, **Pinkment-OH** was synthesized as previously reported^26^ and used to form a **HSA**/**Pinkment-OH** hybrid to illustrate the generality of our encapsulation strategy.

**Fig. 1:**
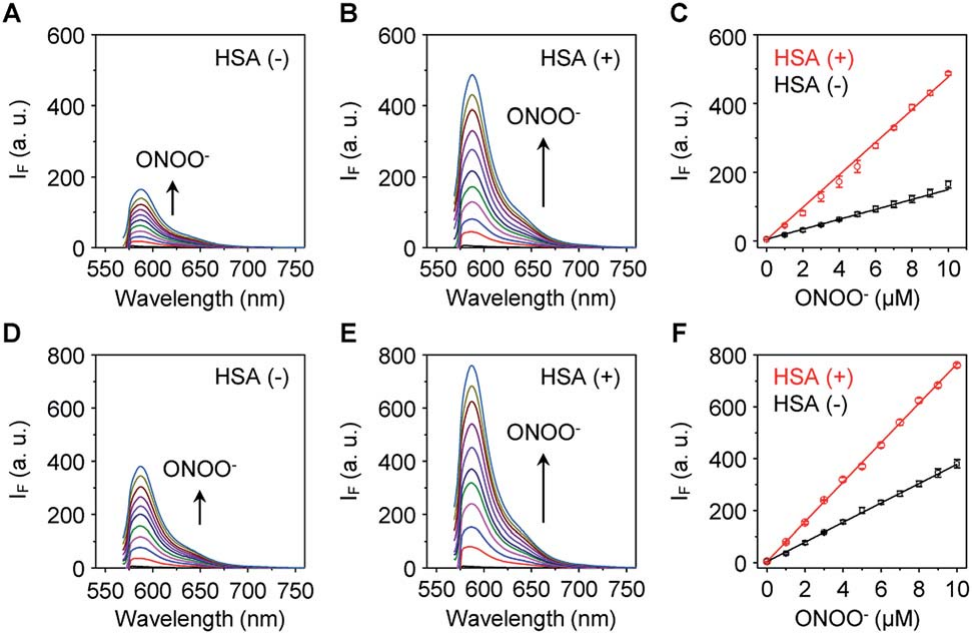
Fluorescence spectra of (A) **Pinkment-OAc** (5 μM) and (B) **HSA**/**Pinkment-OAc** (5/5 μM) with the addition of ONOO^−^ (0–10 μM); phosphate buffered saline – PBS (pH 7.4), *λ*_ex_ = 545 nm. (C) Comparison of the fluorescence intensities of **Pinkment-OAc** (5 μM) and **HSA**/**Pinkment-OAc** (5/5 μM) with the addition of ONOO^−^ (0–10 μM). Fluorescence spectra of (D) **Pinkment-OH** (5 μM) and (E) **HSA**/**Pinkment-OH** (5/5 μM) with the addition of ONOO^−^ (0–10 μM); phosphate buffered saline – PBS (pH 7.4), *λ*_ex_ = 545 nm. (F) Comparison of the fluorescence intensities of **Pinkment-OH** (5 μM) and **HSA**/**Pinkment-OH** (5/5 μM) with the addition of ONOO^−^ (0–10 μM). Error bars represent S. D. (*n* = 3).

The sensitivity of these two probes towards ONOO^−^ was then determined (Fig. 1). Unsurprisingly, **Pinkment-OAc** (Fig. 1A) and **Pinkment-OH** (Fig. 1D) gave rise to a dose dependent increase in fluorescence intensity when exposed to ONOO^−^ in PBS (phosphate buffered saline, pH 7.4) solution. The effect, however, is modest. In contrast, a much stronger response was seen for both **HSA**/**Pinkment-OAc** (Fig. 1B) and **HSA**/**Pinkment-OH** (Fig. 1E). In the presence of ONOO^−^ (10 μM), a 3-fold enhancement in fluorescence intensity was observed for **HSA**/**Pinkment-OAc** (Fig. 1C) whereas **HSA**/**Pinkment-OH** afforded a 2-fold increase in fluorescence intensity (Fig. 1F) when compared to each probe alone. This was taken as an initial indication of the strong affinity of HSA for each **Pinkment** probe. In support of this, the binding constant (*K*_a_) between **Pinkment-OAc** and HSA was measured through isothermal titration calorimetry with HSA (Fig. S1†). As expected, strong binding between **Pinkment-OAc** and HSA was observed with a binding constant of *K*_a_ = 2.83 × 10^5^ L mol^−1^. The quantum yield (*Φ*_F_; relative to quinine sulfate (probe w/o ONOO^−^) and rhodamine B (probe w/ ONOO^−^)) and extinction coefficient (*ε*) of **HSA**/**Pinkment-OAc** before and after addition of ONOO^−^ were determined (Table S1†). Subsequently, the pH-dependence of **Pinkment-OAc** and **HSA**/**Pinkment-OAc** to detect ONOO^−^ was evaluated and compared. Remarkably, a greater ONOO^−^ sensitivity was observed for **HSA**/**Pinkment-OAc** when compared to **Pinkment-OAc** alone over a wide pH range of 3–9 (in particular from pH 5–8) (Fig. S2†).

**Fig. 2:**
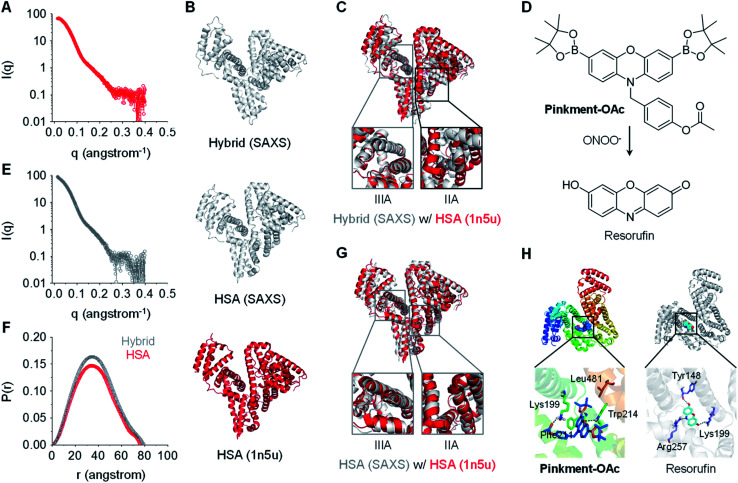
X-Ray scattering patterns and molecular docking of **Pinkment-OAc** to HSA. (A) X-ray scattering pattern of **HSA**. (B) Fitting of atomic models of the hybrid (model refined with scattering data, top), HSA (model refined with scattering data, middle), and the crystal structure of HSA (PDB id 1n5u; bottom). (C) HSA crystal structure superimposed on an atomic model of **HSA**/**Pinkment-OAc** (w/w = 1 : 10) (NMA simulation; crystal structure used to fit the data: PDB id 1n5u). (D) Chemical structure of **Pinkment-OAc** and its reaction with ONOO^−^. (E) X-ray scattering pattern of **HSA**/**Pinkment-OAc** (w/w = 1 : 10). (F) Interatomic distance distribution function, *P*(*r*), of the X-ray scattering patterns of HSA and **HSA**/**Pinkment-OAc** (w/w = 1 : 10). (G) HSA crystal structure superimposed with an atomic model of HSA (NMA simulation; crystal structure used to fit the data: PDB id 1n5u). (H) Proposed binding of **Pinkment-OAc** and resorufin to HSA showing interaction mode between **Pinkment-OAc**, resorufin, and selected amino acid residues of HSA.

In order to gain a greater understanding of the interactions between **Pinkment-OAc** and HSA, small-angle X-ray scattering (SAXS) measurements were carried out. SAXS is a powerful technique that enables conformational changes within biomacromolecules to be observed.^27,28^ The SAXS profile for homogeneous HSA and the **HSA**/**Pinkment-OAc** hybrid (w/w = 1 : 10) is shown in Fig. 2A and E, respectively. To explore the conformational changes of the hybrid, SREFLEX, a hybrid modeling program that systematically combines SAXS data and normal mode analysis (NMA) was used to obtain three-dimensional models of both HSA (Fig. 2B, middle) and the **HSA**/**Pinkment-OAc** hybrid (Fig. 2B, top).^29^ The three-dimensional SAXS model of HSA and **HSA**/**Pinkment-OAc** hybrid were then superimposed onto a resolved HSA atomic structure (PDB entry: 1n5u, Fig. 2B, bottom), respectively, using the program SUPCOMB. The modelling result obtained in this way revealed a significant conformational change of the **HSA**/**Pinkment-OAc** hybrid at the IIA region, and minimal change within the IIIA region with respect to the crystal structure of HSA (Fig. 2C). Conversely, the fitting of the SAXS model of HSA alone with its crystal structure led to good consistency for both the IIA and IIIA regions (Fig. 2G).

We also compared the interatomic distance distribution function (*P*(*r*)) profile for both models (Fig. 2F). The results were consistent with a slightly increased maximum diameter (*D*_max_) for the HSA protein after inclusion of **Pinkment-OAc** to produce **HSA/Pinkment-OAc** (76.4 to 79.3 Å). The SAXS data thus provides support for the formation of a hybrid structure consisting of **Pinkment-OAc** and HSA. These data and the associated modeling also lead us to suggest that **Pinkment-OAc** is mainly bound to the IIA region of HSA. To complement the above studies, a competition assay was performed with a known IIA-region binder (phenylbutazone) and a known IIIA-region binder (ibuprofen). Satisfyingly, it was found that phenylbutazone was significantly better at competitively interrupting the association between **Pinkment-OAc** and HSA compared to ibuprofen (Fig. S3†). This was taken as further evidence that inclusion of **Pinkment-OAc** within HSA occurs predominantly at the IIA region of the protein.

Molecular docking studies were then carried out with the goal of determining how **Pinkment-OAc** is bound within the IIA region of HSA. On the basis of these studies, we propose that **Pinkment-OAc** enters the hydrophobic cavity of the HSA subdomain IIA and is stabilized by a hydrophobic interaction with the amino acid residue, Leu481 (Fig. 2H, left). Meanwhile, the carbonyl oxygen (OAc) and the oxygen on the phenoxazine unit are hydrogen bonded to Lys199 and Trp214, respectively. In addition, the benzene ring of **Pinkment-OAc** is involved in a π-cation interaction with the nitrogen atom of the polar amino acid residue Lys199 and a π–π interaction with Phe211 (Fig. 2H). Taken in concert, these inferred interactions provide a rationale for the inference that **Pinkment-OAc** is tightly bound to HSA at the IIA domain. The modeling studies also revealed that the polar groups of resorufin (the reaction product of **Pinkment-OAc** and ONOO^−^) interact with several polar residues in the IIA domain of HSA. These polar groups include the carbonyl oxygen, phenolic unit, and nitrogen atom of the aromatic core, which stabilize hydrogen bonding interactions with the residues Lys199, Tyr148, and Arg257, respectively (Fig. 2H, right). The resulting strong binding of resorufin within the hydrophobic pocket of HSA would account for the enhanced fluorescence seen when the **HSA**/**Pinkment-OAc** hybrid is exposed to ONOO^−^ (Fig. 1).

**Fig. 3:**
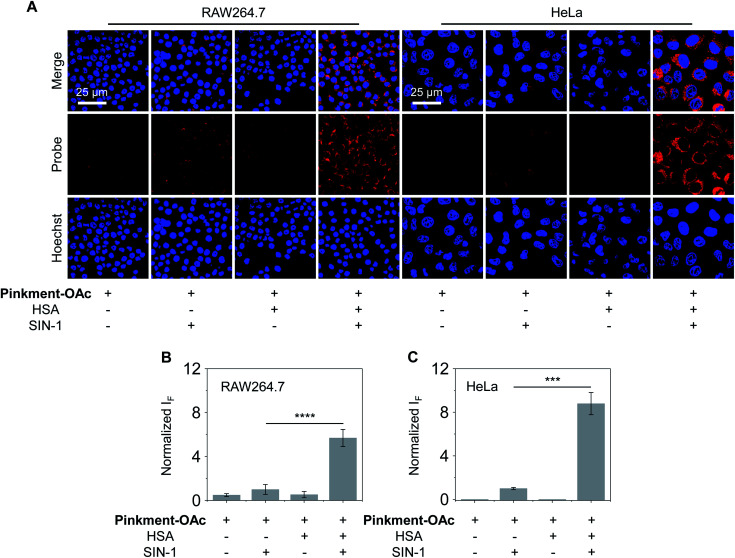
Fluorescence imaging experiments. (A) Confocal images and fluorescence quantification of (B) RAW 264.7 and (C) HeLa cells treated with **Pinkment-OAc** (20 μM, 1% DMSO in PBS) or **HSA**/**Pinkment-OAc** (20/20 μM, 1% DMSO in PBS) with or without added SIN-1 (500 μM). The excitation and emission wavelengths for **Pinkment-OAc** are 559 nm and 580–650 nm, respectively. The cell nuclei were stained with Hoechst 33342. ****P < 0.0001, ***P < 0.001. Error bars represent S. D. (*n* = 3).

As noted above, hybridization between HSA and **Pinkment-OAc** leads to an enhanced fluorescence response in the presence of ONOO^−^. We therefore sought to test whether the **HSA**/**Pinkment-OAc** hybrid could be used to image ONOO^−^*in vitro* and *in vivo*. Towards this end, a macrophage cell line (RAW 264.7) and a human cervical cancer cell line (HeLa) were treated with **Pinkment-OAc** and the **HSA**/**Pinkment-OAc** hybrid. Each cell line was then evaluated with or without SIN-1 (an exogenous agent known to upregulate intracellular ONOO^−^ concentrations).^30^ The cells were then visualized *via* confocal laser-scanning microscopy (CLSM) (Fig. 3). As expected, in the absence of SIN-1, no fluorescence was observed in either cell line; this was true for both free **Pinkment-OAc** and the **HSA**/**Pinkment-OAc** hybrid. Subsequent treatment with SIN-1 led to a remarkable fluorescence increase for **HSA**/**Pinkment-OAc** whereas a minimal fluorescence response was observed for **Pinkment-OAc** alone. Specifically, an overall 5.6-fold (RAW 264.7) and 8.7-fold (HeLa) difference in fluorescence intensity between **HSA**/**Pinkment-OAc** and **Pinkment-OAc** was observed for these two cell lines (Fig. 3B and C). In order to compare the sensitivity of **HSA**/**Pinkment-OAc** to that of **Pinkment-OAc** alone, we used high-content microscopy to quantify the fluorescence intensity of **Pinkment-OAc** and **HSA**/**Pinkment-OAc** in RAW 264.7 cells, both with and without treatment with SIN-1. As shown in Fig. S4,† a 5.4-fold increase in integrated fluorescence intensity for **HSA**/**Pinkment-OAc** was observed when treated with SIN-1 compared to **Pinkment-OAc** alone.

It has been reported that HSA can be actively endocytosed through clathrin-mediated pathway.^31^ To demonstrate that these **HSA**/**Pinkment** probes underwent a similar clathrin-mediated endocytosis, temperature-dependent fluorescence imaging experiments were carried out. In addition, fluorescence imaging experiments were carried out using a known clathrin inhibitor to inhibit clathrin-facilitated endocytosis. A decreased intracellular fluorescence response was observed, when the temperature was decreased to 4 °C (Fig. S5†), suggesting a kinetically controlled cell uptake process. Interestingly, the use of the known clathrin inhibitor chlorpromazine similarly led to a decreased fluorescence response (Fig. S6†), which suggests that **HSA**/**Pinkment-OAc** underwent a clathrin-mediated endocytosis to facilitate cellular uptake of **Pinkment-OAc**. Fluorescence co-localization experiments of **HSA**/**Pinkment-OAc** with a lysosome tracker (Fig. S7†) (Pearson's correlation (*R*_r_) = 0.93), revealed that clathrin-mediated endocytosis resulted in accumulation of **HSA**/**Pinkment-OAc** in the lysosome of cells. This observation is in agreement with a previous report describing the lysosome-localization of HSA-based biomaterials.^32^

To demonstrate that both probe and hybrid displayed minimal toxicity towards cells, an MTS assay was performed using a RAW 264.7 cell line. As expected, little change in the cell viability was observed upon increasing concentrations of **HSA**/**Pinkment-OAc** from 5/5 μM to 80/80 μM (Fig. S8†). These findings further support our suggestion that the HSA hybridization strategy reported here may represent a general and robust approach that could be used to enhance the intracellular sensitivity of small molecule probes *in vitro* without triggering an adverse cellular toxicity response.

**Fig. 4:**
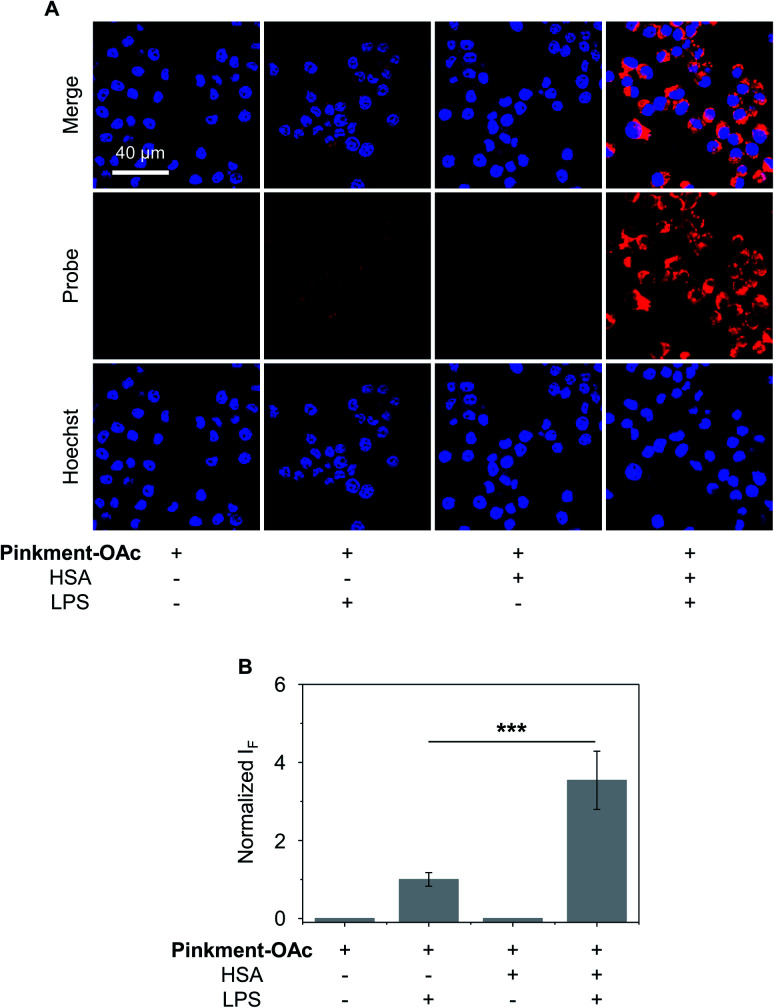
Fluorescence imaging experiments. (A) Confocal images and (B) fluorescence quantification of RAW 264.7 cells treated with **Pinkment-OAc** (20 μM, 1% DMSO in PBS) or **HSA**/**Pinkment-OAc** (20/20 μM, 1% DMSO in PBS) with or without added LPS (lipopolysaccharide, 1 μg mL^−1^). The excitation and emission wavelengths for **Pinkment-OAc** are 559 nm and 580–650 nm, respectively. The cell nuclei were stained with Hoechst 33342. ***P < 0.001. Error bars represent S. D. (*n* = 3).

The over-production of intracellular ONOO^−^ is believed to contribute to the development of a wide variety of inflammatory diseases.^33–35^ Therefore, we turned our attention to testing whether **HSA**/**Pinkment-OAc** and **Pinkment-OAc** could be used to detect endogenous ONOO^−^*in vitro* and *in vivo*. To achieve the imaging of endogenous ONOO^−^*in vitro*, RAW 264.7 cells were treated with lipopolysaccharide (LPS), which is known to induce an inflammatory response with acute upregulation of ONOO^−^.^36^ As shown in Fig. 4, a larger fluorescence increase was observed for **HSA**/**Pinkment-OAc** compared to **Pinkment-OAc** alone (Fig. 4). This again suggests the beneficial effect of the HSA encapsulation strategy for an increased cellular uptake of the probe and enhanced fluorescence imaging of endogenously produced ONOO^−^.

**Fig. 5:**
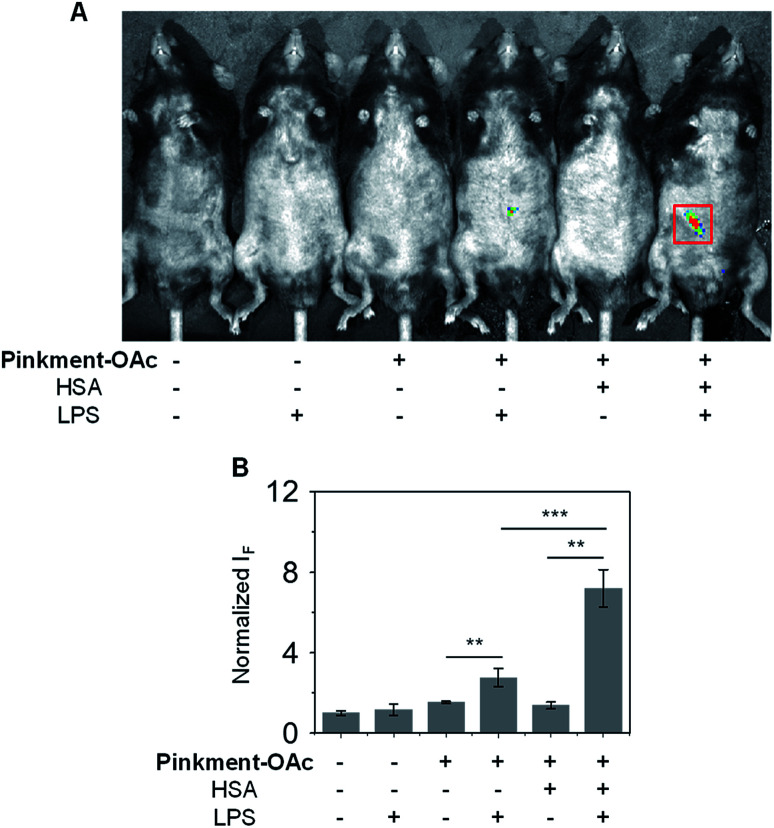
Demonstrating the effectiveness of the **HSA**/**Pinkment-OAc** hybrid for the *in vivo* imaging of ONOO^−^. (A) Fluorescence images and (B) quantification of C57BL/6J mice treated with **Pinkment-OAc** (100 μL, **Pinkment-OAc** = 200 μM in saline) or **HSA**/**Pinkment-OAc** (100 μL, **HSA**/**Pinkment-OAc** = 200/200 μM in saline) in the absence and presence of LPS (200 μL, 2 mg mL^−1^ in saline). **P < 0.01, ***P < 0.001. Error bars represent S. D. (*n* = 3).

Subsequently, an *in vivo* experiment was carried out by treating C57BL/6J mice with LPS. In this way, a mouse model for acute inflammation was established according to the literature.^37,38^ The mice were divided into two groups; one was administered with pure saline intraperitoneally (i.p.) as a control, while the other was injected i.p. with LPS (200 μL, 2 mg mL^−1^) to induce acute inflammation. After 4 h, the mice were anaesthetized, and their abdominal fur was removed. Next, the two groups of mice were treated (*via* i.p. injection) with **DMSO** (control), **Pinkment-OAc**, or **HSA**/**Pinkment-OAc**. Images were acquired using the IVIS spectrum imaging system. In the case of the LPS-treated and saline-treated mice, no fluorescence was observed when injected with DMSO (Fig. 5). However, the LPS-treated mice that were injected with **HSA**/**Pinkment-OAc** produced an approximately 2.7-fold greater increase in fluorescence signal when compared to **Pinkment-OAc** alone (Fig. 5A and B). These *in vivo* experiments provide support for the contention that the present **HSA**/**Pinkment-OAc** hybrid approach allows ONOO^−^ production to be monitored effectively *in vivo* and with greater sensitivity than provided by the constituent probe (**Pinkment-OAc**).

## Conclusions

In this work, we have demonstrated the viability of a simple protein-based hybridization strategy, that involves incorporation of an insoluble ONOO^−^ fluorescent probe (**Pinkment-OAc**) with HSA. The resulting **HSA**/**Pinkment-OAc** hybrid was characterized using SAXS analyses in conjunction with molecular docking studies. In solution, the **HSA**/**Pinkment-OAc** hybrid produced an enhanced fluorescence response towards ONOO^−^ compared to the **Pinkment-OAc** probe alone. More importantly, the **HSA**/**Pinkment-OAc** hybrid permitted improved ONOO^−^ detection in RAW 264.7 and HeLa cells relative to the free **Pinkment-OAc** probe. This enhancement is ascribed to improved cell permeability. In addition, our hybrid strategy was effective for the *in vivo* imaging of acute inflammation in LPS-treated mice, where again an improved performance was seen compared to the probe alone. On the basis of the present findings, we suggest that this approach represents a simple and general strategy to overcome the issues of low solubility and poor cell permeability that currently limit the use of hydrophobic imaging agents *in vivo*.

## Conflicts of interest

There are no conflicts to declare.

## Supplementary Material

SC-011-C9SC03961A-s001
