# Supplementary material for: Protein encapsulation: a new approach for improving the capability of small-molecule fluorogenic probes
Source: Chem Sci. 2019 Nov 27;11(4):1107–13. doi: 10.1039/c9sc03961a (PMC8145178; doi:10.1039/c9sc03961a)
Supplement: SC-011-C9SC03961A-s001 [file SC-011-C9SC03961A-s001.pdf]

Electronic Supplementary Material (ESI) for Chemical Science.
This journal is © The Royal Society of Chemistry 2019

Protein Encapsulation: A New Approach for Improving the Capability of Small-Molecule Fluorogenic Probes

Hai-Hao Han,[1,3,a] Adam C. Sedgwick,[2,4a] Ying Shang,[1,a] Na Li,[5] Tingting Liu,[3] Bo-Han Li,[3] Kunqian Yu,[3] Yi Zang,[3] James T. Brewster II,[4] Maria L. Odyniec,[2] Maria Weber,[2] Steven D. Bull,[2] Jia Li,[3,*] Jonathan L. Sessler,[4,6*] Tony D James,[2,7,*] Xiao-Peng He[1,*] and He Tian[1]

[1]Key Laboratory for Advanced Materials and Joint International Research Laboratory of Precision Chemistry and Molecular Engineering, Feringa Nobel Prize Scientist Joint Research Center, School of Chemistry and Molecular Engineering, East China University of Science and Technology, 130 Meilong Road, Shanghai 200237, People's Republic of China
[2]Department of Chemistry, University of Bath, Bath, BA2 7AY, UK
[3]National Center for Drug Screening, State Key Laboratory of Drug Research, Shanghai Institute of Materia Medica, Chinese Academy of Sciences, 189 Guo Shoujing Rd., Shanghai 201203, P. R. China
[4]Department of Chemistry. University of Texas at Austin, 105 East 24th Street A5300, Austin, Texas 78712-1224, United States
[5] National Facility for Protein Science in Shanghai, Zhangjiang Laboratory, Shanghai 201210, P. R. China
[6]Center for Supramolecular Chemistry and Catalysis, and Department of Chemistry, Shanghai University, 99 Shang-Da Road, Shanghai 200444, China
[7]Lead contact
[a]Equal contribution

Email:
t.d.james@bath.ac.uk (T.D.J.)
xphe@ecust.edu.cn (X.P.H.)
sessler@cm.utexas.edu (J.L.S.)
jli@simm.ac.cn (J.L.)

Table of Contents

1. **General methods** S3

2. **Fluorescence "Turn on" mechanism** S6

3. **Binding studies with HSA** S6

4. **Cellular imaging and cell viability assays** S8

5. **References** S11

1. General methods

General remarks.

Chemicals were used as received unless otherwise indicated. All other reagents are of analytical purity and used without further treatment. Solvents used are of analytical grade. UV-absorbance spectra were carried out on a Varian Cary 500 spectrophotometer. Fluorescence spectra were recorded on a Varian Cary Eclipse fluorescence spectrophotometer. Isothermal titration calorimetric (ITC) measurement was performed with Affinity ITC. The fluorescence cell images were recorded by an Operetta high content imaging system (Perkinelmer, US) or a confocal laser scanning microscopy (Olympus, Japan; Leica TCS SP8, Germany).

Preparation of HSA/probe hybrids.

Stock solution of **Pinkment-OAc** and **Pinkment-OH** (1 mM) was prepared in DMSO. Stock solution of HSA (0.5 mM) was prepared in phosphate buffered saline (PBS, 0.01 M, pH 7.4). **HSA/Pinkment-OAc** and **HSA/Pinkment-OH** (5/5 µM) hybrid solution was prepared by simply mixing the probe with different concentrations of HSA, and the resulting mixture was incubated for 5 min to allow for sufficient host-guest binding.

Fluorescence microscopy.

The fluorescence measurements were carried out with a path length of 5 mm and an excitation wavelength of 545 nm by scanning the spectra between 545 nm and 800 nm. The bandwidth for both excitation and emission spectra was 5 nm. Unless otherwise mentioned, all the spectra were recorded at 25 °C.

Small Angle X-ray Scattering (SAXS) experiment

Small-angle X-ray scattering (SAXS) experiments were performed at beamline BL19U2 of the National Facility for Protein Science Shanghai (NFPS) at the Shanghai Synchrotron Radiation Facility (SSRF). The wavelength, λ, of the X-ray radiation was set at 1.033 Å. Scattered X-ray intensities were collected using a Pilatus 1M detector (DECTRIS Ltd). The sample-to-detector distance was set such that the detecting range of momentum transfer [$q = 4\pi \sin\theta/\lambda$, where 2θ is the scattering angle] of the SAXS experiments was 0.01-0.40 $Å^{-1}$. To reduce the radiation damage, a flow cell made of a cylindrical quartz capillary with a diameter of 1.5 mm and a wall thickness of 10 µm was used. The protein HSA for the SAXS measurement was prepared at 10 µM in PBS (10 mM, pH 7.4) in the presence or absence of 10 molar equivalents of Pinkment-OAc. The scattering profiles for a matched buffer were subtracted from that of the protein. For each measurement, the SAXS data were collected as 20 x 1 sec exposures. The 2-D scattering images were converted to 1-D SAXS curves through azimuthally averaging after applying a solid angle correction and then normalizing with the intensity of the transmitted X-ray beam, using the software package BioXTAS RAW.[1] Background scattering was

subtracted using the PRIMUS program in the ATSAS software package.[2] The conformational change of the hybrid site was observed using the SREFLEX program within the ATSAS software package.[3] The SUPCOMB program in the ATSAS software package was used to superimpose a three-dimensional SAXS structure onto a literature HSA atomic structure as detailed in the main text.[4]

Protein and ligand preparation

The HSA-drug complex crystal structure 2BXI (2.5 Å) obtained from the RCSB PDB (Protein Data Bank)[5] was selected because the ligand azapropazone molecule (AZQ) in that structure is similar to **Pinkment-OAc**. Protein preparation was performed with the Protein Preparation Wizard panel in Maestro (Schrödinger, LLC, New York, NY, USA).[6] The HSA structure 2BXI was prepared by assigning charges and bond order, adding missing hydrogen atoms and disulfide bonds, and removing waters with fewer than 3 H-bonds, followed by structure minimization. Subsequently, **Pinkment-OAc** and Resorufin were prepared using the LigPrep module in Maestro package and the OPLS_2005 force field under a target pH of 7.0 ± 2.0.

Induced fit docking

The center of a docking box was defined according to the centroid of the workspace ligand AZQ, followed by grid generation using a 15 Å enclosing box. Molecular docking simulations were performed using the Induced Fit Docking (IFD) protocol in the Maestro package.[7] The IFD approach allows for dockings of a flexible ligand with flexible side chains of a protein (the backbone residues of the protein were kept rigid). As a result, twenty binding poses ranked according to the Glide score produced, from which the poses with docking score values of -8.786 and -9.704 were selected as the best binding arrangements of **Pinkment-OAc** and resorufin, respectively. PyMOL viewer (http://www.pymol.org/) was used to visualize binding poses and to generate molecular images.

Cell culture

RAW264.7 and HeLa cells were maintained in a Dulbecco's Modified Eagle's Medium (Invitrogen, Carlsbad, CA, USA) supplemented with 10% fetal bovine serum (Gibco, Gland Island, NY, USA) in a humidified atmosphere of 5% CO_2 and 95% air at 37°C and split when the cells reached 90% confluency.

High-content fluorescence imaging

Cells were seeded on a black 96-well microplate with an optically clear bottom (Greiner bio-one, Germany) overnight. The cells were incubated with **Pinkment-OAc** (20 µM) or **HSA/Pinkment-OAc** (20/20 µM) for 30 min, followed by incubation with SIN-1 (500 µM) for 30 min. Then, cells were washed with PBS (phosphate buffered saline) three times. The fluorescence images were recorded using an Operetta high content imaging system (Perkin Elmer, USA) at an excitation wavelength of 520–550 nm and an emission wavelength of 580–

650 nm. Data were quantified and plotted using the manufacturer's Columbus Analysis system (Perkin Elmer, USA).

Confocal laser scanning microscopy

Cells cultured in growth medium supplemented with 10% fetal bovine serum were plated onto 24-well microplates. Cells were maintained in a humidified atmosphere of 5% CO_2 and 95% air at 37°C overnight. Then, cells were incubated with **Pinkment-OAc** (20 µM) or **HSA/Pinkment-OAc** (20/20 µM) for 30 min, followed by incubation with SIN-1 (500 µM) for 30 min. The cells were then rinsed with warm PBS and fixed using 4% paraformaldehyde for 20 min. The cell nuclei were stained with Hoechst 33342 (5 µg mL^{-1}) at room temperature for 5 min. After three rinses with PBS, the fluorescence was detected and photographed using a confocal laser scanning microscopy (Olympus, Japan; Leica TCS SP8, Germany).

Cell viability assay

Cells were plated on 96-well plates in growth medium. After 24 h, cells were treated with **Pinkment-OAc** and **HSA**/**Pinkment-OAc** at different concentrations for 24 h. Then, the cell viabilities were determined through a standard MTS cell proliferation assay.

***In Vivo* Imaging**

C57BL/6J mice were divided into two groups, the first group was given an intraperitoneal (i.p.) injection of saline (200 µL) as a control, the second group was injected i.p. with lipopolysaccharide (200 µL, 2 mg mL^{-1} in saline, Sigma–Aldrich, USA) for 4 h. The mice were anesthetized and the abdominal fur was removed using a razor. They were then injected i.p. with DMSO, **Pinkment-OAc** (100 µL, 200 µM in saline), or **HSA/Pinkment-OAc** (100 µL, 200/200 µM, in saline). Whole body images were acquired within 30 min using an IVIS Spectrum imaging system (Perkin Elmer, USA). The imaging parameters were set as follows: Ex. 550 nm, Em. 580-620 nm.

2. Fluorescence "turn-on" mechanism

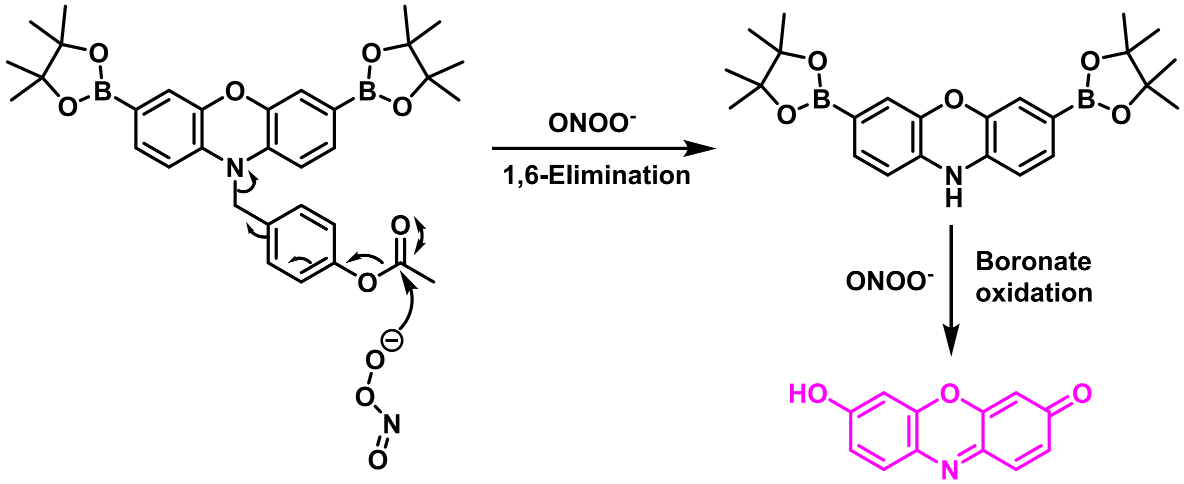

Scheme S1 – Proposed fluorescence turn-on mechanism for **Pinkment-OAc** when treated with peroxynitrite ($ONOO^{-}$)

3. Binding studies with HSA

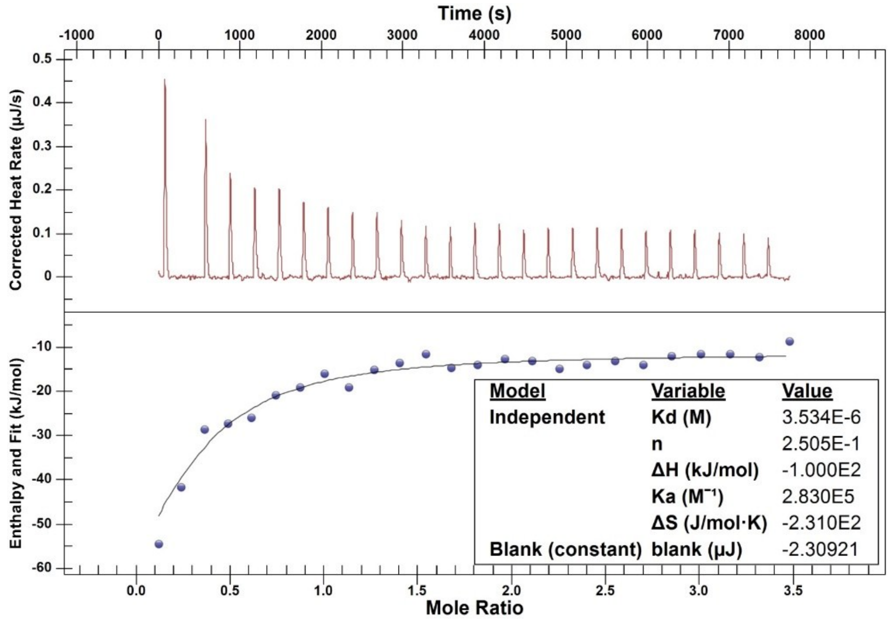

Fig. S1 – Isothermal titration calorimetric (ITC) analysis of the binding constant between **Pinkment-OAc** (10 µM) and HSA (100 µM) at 25 °C in H_2O (containing 1% DMSO, v/v).

Table S1 – Photophysical parameters of **Pinkment-OAc** and **HSA/Pinkment-OAc** before and after addition of $ONOO^-$.

Group	Φ_F	ε_{max}
**Pinkment-OAc**	0.0574 (λ_{em} = 360 nm)	29168.33 (λ_{em} = 360 nm)
**Pinkment-OAc** w/ $ONOO^-$	0.105 (λ_{em} = 545 nm)	10933.33 (λ_{em} = 545 nm)
**HSA/Pinkment-OAc**	0.0797 (λ_{em} = 360 nm)	26008.33 (λ_{em} = 360 nm)
**HSA/Pinkment-OAc** w/ $ONOO^-$	0.356 (λ_{em} = 545 nm)	8086.67 (λ_{em} = 545 nm)

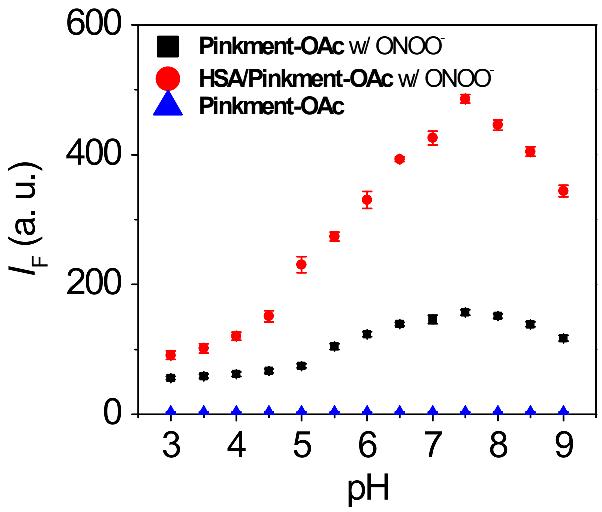

Fig. S2 – Plotting the fluorescence change of **Pinkment-OAc** (5 μM), **HSA/Pinkment-OAc** (5/5 μM), and **HSA/Pinkment-OAc** (5/5 μM) in the presence of $ONOO^-$ (10 μM) as a function of pH (3-9). The measurements were carried out in PBS (λ_{ex} = 545 nm).

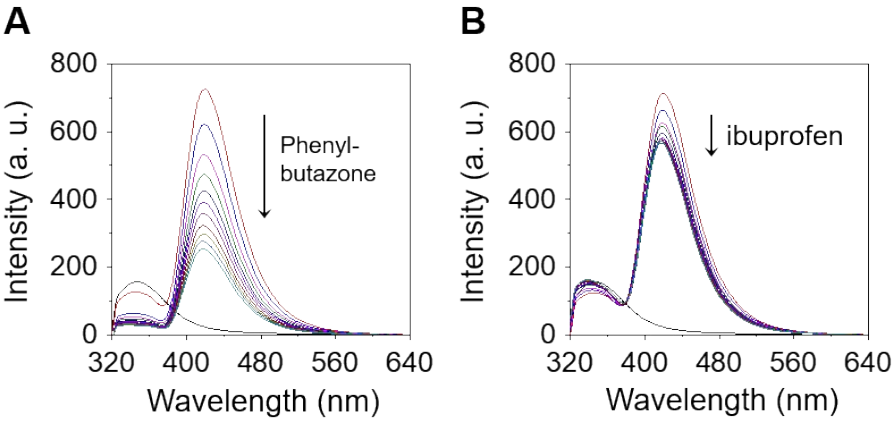

Fig. S3 – Changes in fluorescence emission intensity (λ_{ex} = 295 nm, pH 7.4) seen when HSA (4 μM) is first treated with **Pinkment-OAc** (2 μM) and then titrated with (A) phenylbutazone (0-100 μM) and (B) ibuprofen (0-100 μM).

4. Cellular imaging and cell viability assays

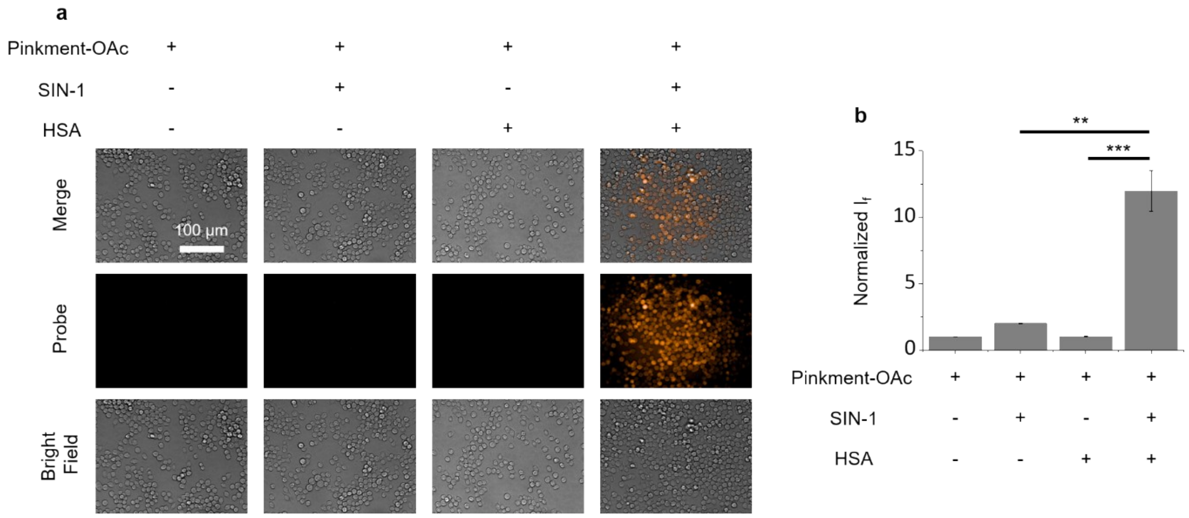

Fig. S4 – Fluorescence imaging (a) and quantification (b) of RAW264.7 cells after incubation with **Pinkment-OAc** (20 µM, 1% DMSO in PBS) or **HSA/Pinkment-OAc** (20/20 µM, 1% DMSO in PBS) in the absence and presence of SIN-1 (500 µM, a $ONOO^-$ donor). The excitation and emission wavelengths for **Pinkment-OAc** are 520–550 nm and 580–650 nm, respectively. Scale bar = 100 µm. Error bars represent the s.d. (n = 3).

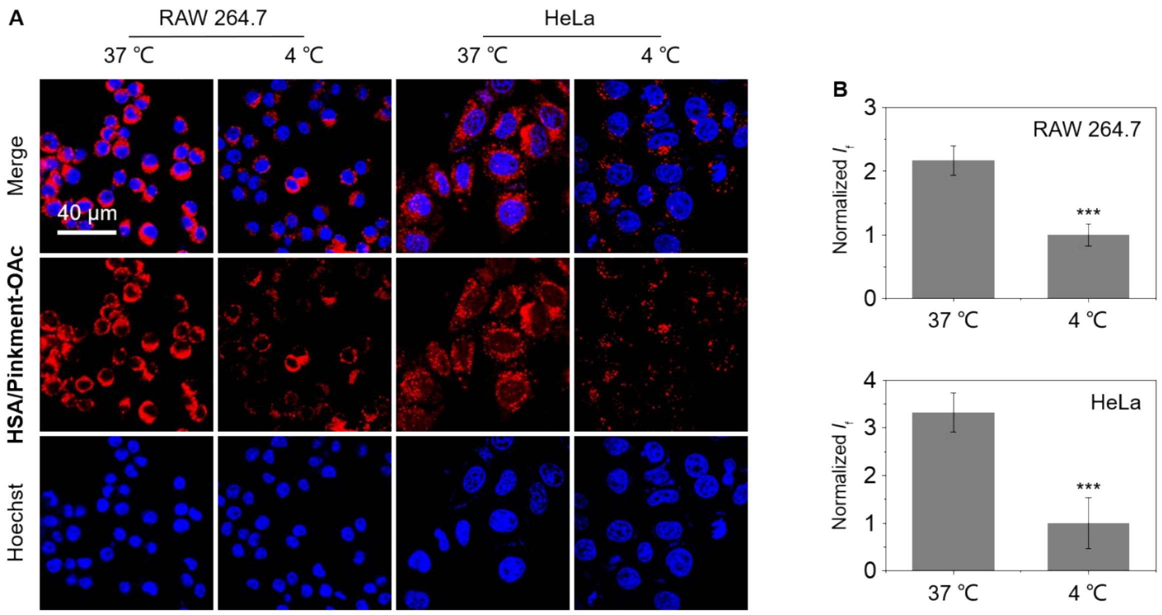

Fig. S5 – Fluorescence imaging experiments. (A) Confocal images and (B) fluorescence quantification of RAW264.7 and HeLa cells treated with **HSA/Pinkment-OAc** (20/20 µM, 1% DMSO in PBS) with different temperature. SIN-1 (500 µM) was then added to elicit a fluorescent response. The excitation and emission wavelengths for **Pinkment-OAc** are 559 nm and 580–650 nm, respectively. The cell nuclei were stained with Hoechst 33342. ***P<0.001. Error bars represent s.d. (n = 3).

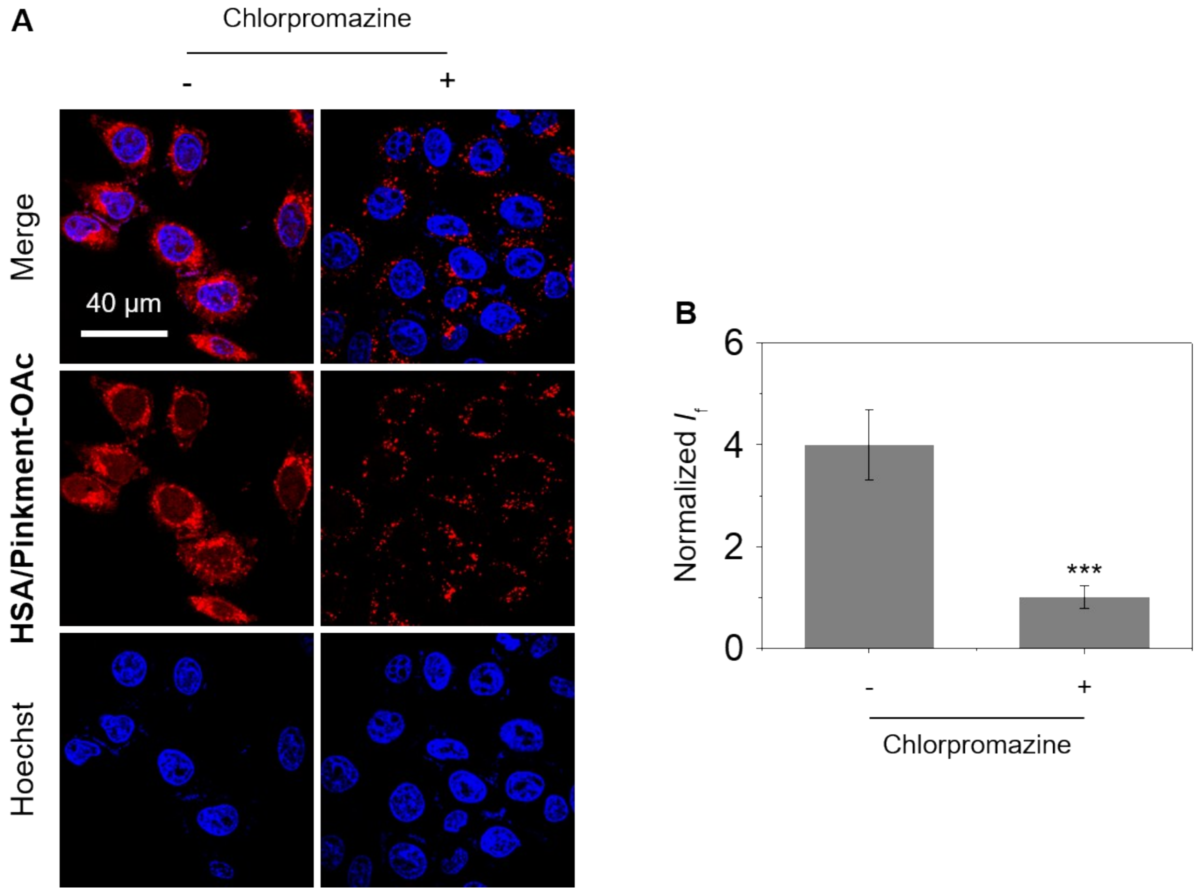

Fig. S6 – Fluorescence imaging experiments. (A) Confocal images and (B) fluorescence quantification of HeLa cells treated with **HSA/Pinkment-OAc** (20/20 μM, 1% DMSO in PBS) before and after addition of chlorpromazine (a clathrin inhibitor; 30 μM). SIN-1 (500 μM) was then added to elicit a fluorescent response. The excitation and emission wavelengths for **Pinkment-OAc** are 559 nm and 580–650 nm, respectively. The cell nuclei were stained with Hoechst 33342. ***P<0.001. Error bars represent s.d. (n = 3).

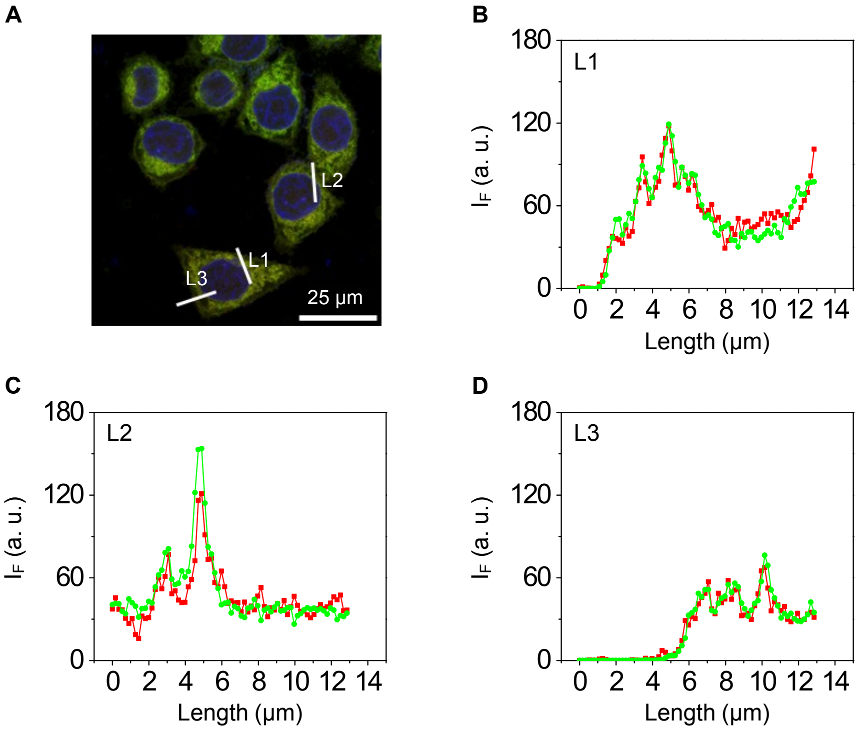

Fig. S7 – (A) Fluorescence co-localization of **HSA/Pinkment-OAc** (20/20 µM, 1% DMSO in PBS) with LysoTracker® Deep Red (a lysosome tracker, 500 nM) in HeLa cells. (B, C, D) Fluorescence quantification of selected cellular regions (white solid lines in panel A) of **HSA/Pinkment-OAc** (20/20 µM, 1% DMSO in PBS, red dots) and LysoTracker® Deep Red (500 nM, green dots) HeLa cells. SIN-1 (500 µM) was then added to elicit a fluorescent response. Excitation wavelengths for **Pinkment-OAc** and LysoTracker® Deep Red are 559 nm and 638 nm, respectively. Emission wavelengths for **Pinkment-OAc** and LysoTracker® Deep Red are 580–650 nm and 650-670 nm, respectively. Error bars are S. D. (n = 3).

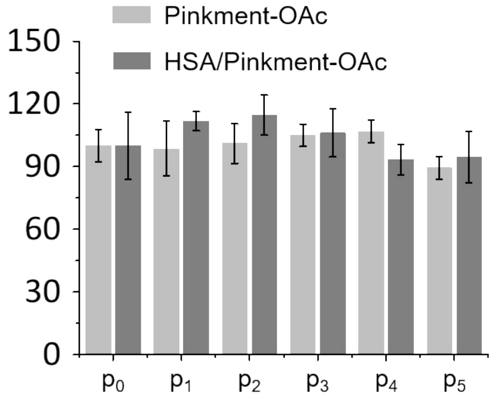

Fig. S8 – Cell viability of RAW264.7 cells after treatment with different concentrations of **Pinkment-OAc** and **HSA/Pinkment-OAc** as determined by MTS assay. (p_0 = Blank, p_1 = **Pinkment-OAc** (5 µM), **HSA/Pinkment-OAc** (5/5 µM), p_2 = **Pinkment-OAc** (10 µM), **HSA/Pinkment-OAc** (10/10 µM), p_3 = **Pinkment-OAc** (20 µM), **HSA/Pinkment-OAc** (20/20 µM), p_4 = **Pinkment-OAc** (40 µM), **HSA/Pinkment-OAc** (40/40 µM), p_5 = **Pinkment-OAc** (80 µM), **HSA/Pinkment-OAc** (80/80 µM)). Error bars represent the s. d. (n = 3).

5. References

1. J. B. Hopkins, R. E. Gillilan and S. Skou, *J. Appl. Crystallogr.*, 2017, **50**, 1545-1553.
2. M. V. Petoukhov, D. Franke, A. V. Shkumatov, G. Tria, A. G. Kikhney, M. Gajda, C. Gorba, H. D. Mertens, P. V. Konarev and D. I. Svergun, *J. Appl. Crystallogr.*, 2012, **45**, 342-350.
3. A. Panjkovich and D. I. Svergun, *Phys. Chem. Chem. Phys.*, 2016, **18**, 5707-5719.
4. M. B. Kozin and D. I. Svergun, *J. Appl. Crystallogr.*, 2001, **34**, 33-41.
5. H. M. Berman, J. Westbrook, Z. Feng, G. Gilliland, T. N. Bhat, H. Weissig, I. N. Shindyalov and P. E. Bourne, *Nucleic Acid Res.*, 2000, **28**, 235-242.
6. K. M. Elokely and R. J. Doerksen, *J. Chem. Information Modeling*, 2013, **53**, 1934-1945.
7. W. Sherman, H. S. Beard and R. Farid, *Chem. Biol. Drug Design*, 2006, **67**, 83-84.
